# The Mediating Effect of Presenteeism on the Relationship between Emotional Labor and Work Engagement of Coaches for Disability Sports

**DOI:** 10.3390/ijerph20020919

**Published:** 2023-01-04

**Authors:** Eun-Chul Seo, Young-Kyun Sim, Inwoo Kim, Jae-Pil Seo, Min-Seong Ha, Song-Eun Kim

**Affiliations:** 1 Department of Physical Education, Wonkwang University-Iksan, 460 Iksan-daro, Iksan 54538, Republic of Korea; 2 Department of International Sports, Dankook University-Chungcheongnam-do, 119 Dandae-ro, Dongnam-gu, Cheonan-si 31116, Republic of Korea; 3 Department of Sports Culture, College of the Arts, Dongguk University-Seoul, 30 Pildong ro 1 gil, Jung gu, Seoul 04620, Republic of Korea; 4 Department of Physical Education, Woosuk University-Jeollabuk-do, 443, Samnye-ro, Samnye-eup, WanjuGun 55338, Republic of Korea; 5 Department of Social Physical Education, Soonchunhyang University, 22-9, Soonchunhyang-ro, Sinchang-myeon, Asan-si 31538, Republic of Korea

**Keywords:** emotional labor, disability sports coach, presenteeism, work engagement

## Abstract

The aim of present study was to examine the mediating effect of presenteeism on the relationship between emotional labor and work engagement of coaches for disability sports. A total of 198 coaches in Korea participated in this study. Participants responded a survey measuring emotional labor, presenteeism, and work engagement. After analyzing the correlation between each variable, the mediation model was verified through structural equation model analysis. The results reveal that emotional labor of coaches for disability sports positively predicted their work engagement, but negatively predicted presenteeism. In addition, the participants’ presenteeism can have a negative effect on their work engagement. Moreover, completing work in presenteeism was found to mediate the relationship between the deep acting and surface acting in emotional labor and work engagement of disability sports coaches.

## 1. Introduction

Sports coach is widely known to be a stressful and demanding profession. Coaches usually suffer from a variety of stressors, including irregular working hours, precarious employment, and pressure of results in athletes’ performance and competition [1]. These stressors can induce negative emotional states of the coaches, which can be transmitted to the athletes and negatively affect their psychological aspects such as motivation and self-confidence [2]. Thus, for psychological conditioning of athletes, coaches need to control their negative emotions even in stressful situations.

Controlling emotions and emotional expression for job performance is called emotional labor. Hochschild [3] defined in her original study the term ‘emotional labor’ as “the management of feeling to create a publicly observable facial and bodily display” (p. 7). Hochschild [3] and Grandey [4] explained emotional labor by dividing it into surface acting and deep acting. According to them, although these two types of acting are emotional labor to express the emotions required by the job environment, the deep acting is an effort to feel the emotions required by the job, and the surface acting is an effort to express those emotions. Thus, an individual sometimes hides or deceives emotions (surface acting) to express the emotions required in his or her workplace, and sometimes tries to actually feel those emotions (deep acting), which is called emotional labor. Since then, many studies have been conducted on the outcome variables of the two types of emotional labor. Hülsheger and Schewe [5] revealed in their meta-analysis study that surface acting is positively related to personal ill-being, but negatively related to job-related well-being, but consistent implications for the relationship between deep acting and the outcome variables have not been verified.

Emotional labor is essential in sports coaching due to the inherent dynamics of a sports setting. Coaches experience extreme emotional changes, from great joy to severe frustration, depending on the game situation, but they must control their emotional expressions because their expressions affect the psychological states and performance of the athletes [6]. Also, sports coaching, like teaching, is a job that requires more emotional labor because physical interaction with others is very important in this job [7]. Such high level of emotional labor that occurs in the workplace of sports coaches can seriously affect their personal outcomes. For example, expressing demanded emotions rather than their actual emotions can lower the coaches’ general well-being and coaching effectiveness [8]. Moreover, coaches with a high level of surface acting can perceive job stress more negatively and may experience burnout [9].

Research on the relationship between emotional labor and burnout in sports coaches have been conducted continuously. In general, surface acting was positively related to coach’s burnout, but deep acting was negatively related to it [5]. According to this study, surface acting induces a high level of psychological cost in the process of suppressing negative emotions and expressing emotions required by the situation, while deep acting is a process of modifying feelings before expressing emotions, so there is little psychological cost. In this context, it is possible to assume that the psychological cost involved in surface acting will affect not only the burnout of sports coaches but also other job-related variables. Since these variables affect the performance and conditioning of athletes, it is necessary to pay attention to the effect of emotional labor on job-related variables of coaches.

Implications on the emotional labor of sports coaches have been derived through various studies, but studies related to coaches for disabled sports are hard to find. Coaches in disability sports may have to put more effort into emotional labor than general sports coaches. Caring for people with disabilities is a highly emotional service task, so it can cause more emotional labor than working with general individuals [10]. Also, most coaches in disability sports are non-disabled, so there is a general lack of understanding of disability [11], which means that coaches have to make more emotional effort into generating positive emotions in the athletes with disabilities. Thus, this study aimed to investigate the effect of the emotional labor of coaches in disability sports on their job-related factors.

Work engagement defined as a positive and fulfilling state of mind related to work consists of three sub-factors, including vigor, dedication, and absorption [12], and it is considered one of the job-related factors that can be affected by emotional labor. Vigor means strong energy of the body and mind for work, dedication refers to the emotional state such as enthusiasm and inspiration for work, and absorption means cognitive state that is fully focused and involved in work [12]. Employees’ high vigor and dedication enable them to overcome various obstacles in the job performance, and those with high absorption are able to stay away from distractions and fully focus on their work [13]. The importance of work engagement has been emphasized because it has a positive effect on both job performance and job satisfaction levels of employees.

Previous studies on emotional labor and work engagement generally agree that surface acting has a negative effect on work engagement, while deep acting has a positive effect [7,14]. It means that to increase passion and concentration on work, it would be desirable to align the required emotions with one’s real feelings rather than expressing fake emotions. According to the studies, emotional dissonance due to surface acting causes emotional exhaustion, which leads to diminish work engagement of employees [15,16,17]. On the other hand, deep acting has positive effect on the motivation of employees to work and improves work engagement by harmonizing their emotions and expressions [7,14,17]. Thus, we hypothesized the following:

**Hypothesis** **1.** *The emotional labor of the coaches for disability sports predicts their work engagement.*

Despite the generally agreed findings on emotional labor and work engagement, conflicting research results have also been reported. Several studies have shown that emotional labor has a positive relationship with work engagement without dividing it into sub-dimensions [7,18,19]. Öngöre [20] identified that surface acting is also a kind of emotional efforts of employees that is positively related to vigor for work. With Sports coaches’ work engagement is related to coaching performance [21], several studies conducted and reported that coaches’ emotional labor is one of the causes of decreasing work engagement for coaching and coaching ability [22,23]. Such conflicting implications may suggest the possibility of another variable that can affect the relationship between emotional labor and work engagement.

Sports coaches’ emotional labor and work engagement may also be related to ‘presenteeism’. Presenteeism refers to a state in which work is not performed properly as it is physically present but does not exist functionally or spiritually [24]. There are two dimensions in presenteeism: completing work related to physical causes and avoiding distraction related to psychological causes [25]. Job-related stress and excessive workload are known to be the main causes of presenteeism [26,27], and physical and mental exhaustion was also found to cause presenteeism [27,28]. Presenteeism is known to negatively affect the productivity or effectiveness of work. There are research results showing that presenteeism due to these factors, that is, performing work under physically and mentally exhaustion, causes greater losses than not working at all [29,30].

As the impact of presenteeism on work has been revealed, it is being actively studied in the service industry where emotional demands are high while directly dealing with people. Sports coaches, with high emotional demands, often must instruct athletes in training or running games even when they are emotionally exhausted through emotional labor. This means that the emotional labor of sports coaches can cause their presenteeism. However, in the sports field, there are some studies on presenteeism in that athletes compete with health problems such as injuries [31,32], but there are few studies on the presenteeism of coaches. Based on these findings, we hypothesized the following:

**Hypothesis** **2.** *The emotional labor of the coaches for disability sports predicts their presenteeism.*

Presenteeism can also influence work engagement. A study conducted on employees in medical clinics found that presenteeism had a negative effect on work engagement and job satisfaction [33]. Also, it is identified that presenteeism-related health conditions negatively affected work engagement in production workers [34]. According to the studies, presenteeism weakens the ability of workers to recover from stress or fatigue at workplace, which can negatively affect the work engagement [33,34]. It might mean that presenteeism of sports coaches can also negatively affect work engagement by lowering their resilience. Thus, we hypothesized the following:

**Hypothesis** **3.** *The presenteeism of the coaches for disability sports predicts their work engagement.*

In summary, sports coaching is a job with heavy emotional labor, and coaching for disabled sports may have higher emotional demands. Emotional labor affects coaches’ work engagement, which is one of the important variables for effective coaching such as athletes’ performance and conditioning. However, since the relationship between the emotional labor and work engagement has not been clarified, it is necessary to verify the mediating effect of presenteeism, which has been found to be related to these two variables. Therefore, we hypothesized the following:

**Hypothesis** **4.** *The presenteeism of the coaches for disability sports mediate the relationship between the emotional labor and work engagement.*

In other words, the purpose of this study was to verify the mediating effect of presenteeism on the relationship between emotional labor and the work engagement of coaches for disability sports. Through this, we intend to provide useful implications that can contribute to enhancing the coaching effectiveness for improving and conditioning the performance of athletes with disabilities.

## 2. Methods

### 2.1. Participants

A total of 205 disability sports coaches registered with the Korean Paralympic Committee were selected using a convenient sampling method to participate in this study. After excluding seven data that responded incompletely, a total of 198 data were used for the analysis. The specific characteristics of the participants can be found in Table 1.

### 2.2. Study Procedure

Since this study is a cross-sectional study, the survey was conducted once by directly visiting the participants. Prior to the visit, consent to participate in the study was obtained verbally, and a questionnaire was distributed after obtaining a signature on the study explanation and consent form. To minimize common method bias, a 5-min break was taken before answering the questions on the dependent variable, and no personally identifiable information was collected to ensure anonymity. Additionally, to reduce an acquiescence effect, we distributed positive and negative questions, and mixed the order of the items, and these steps was applied according to the suggestion of Podsakoff, MacKenzie, and Podsakoff [35]. This study was conducted in accordance with the principles set forth in the Declaration of Helsinki, and all procedures involving human participants were approved by the Institutional Review Board of Dongguk University (DUIRB-202206-18).

### 2.3. Measures

Emotional labor. The Emotional Labor Scale (ELS) developed by Grandey [4] and translated into Korean by LEE [36] was used to measure the level of the emotional labor of participants. The ELS consists of a total of eight items, including four items for deep acting and four items for surface acting, and was measured on a 5-point Likert scale. The validity of this questionnaire was verified through previous studies [22], and the results of the confirmatory factor analysis conducted in this study revealed that the model fit was acceptable (*χ*^2^ = 35.8, df = 19, TLI = 0.977, CFI = 0.984, RMSEA = 0.065, SRMR = 0.040).

Presenteeism. To measure the presenteeism of the participants, Stanford Presenteeism Scale (SPS) developed by Turpin et. al. [37] and translated into Korean by LEE [38] was used. The SPS consists of a total of 10 items, including five items for completing work and five items for avoiding distraction, and was measured on a 5-point Likert scale. As a result of confirmatory factor analysis, the construct validity of SPS was verified with the fit indices (*χ*^2^ = 16.3, df = 13, TLI = 0.994, CFI = 0.996, RMSEA = 0.027, SRMR = 0.025).

Work engagement. Participants work engagement was measured using a questionnaire developed by Schaufeli et. al. [12], and translated into Korean by LEE, Kim, and Shin [39]. The questionnaire consists of a total of 15 items, including five items for vigor, five items for dedication, and five items for absorption, and was measured on a 5-point Likert scale. According to the result of the confirmatory factor analysis, three items with overlapping factor loading were deleted. The fit indices of 12 items were acceptable (*χ*^2^ = 118, df = 51, TLI = 0.935, CFI = 0.949, RMSEA = 0.079, SRMR = 0.059).

To verify the convergent and discriminant validity and the internal consistency, composite reliability (CR), average variance extracted (AVE), and Cronbach’s alpha (α) of each variable were calculated. The reliability and convergent validity of all variables were found to be acceptable as shown in Table 2, and since the minimum AVE value was 0.835 and the maximum square value of the correlation coefficient was 0.408, the discriminant validities for all variables were also confirmed.

### 2.4. Analysis

For the analysis, Jamovi 2.3 and AMOS 23.0 programs were used, and the statistical significance level was set to 0.05. First, a descriptive statistical analysis was conducted to understand the participants’ demographic characteristics, including frequency analysis and test for normality. The construct validity of the measurements was verified by confirmatory factor analysis by the maximum likelihood method, and the relationship between variables was examined through correlation analysis. After verifying the validity of the measurement model by applying the two-step analysis method of Anderson & Gerbing [40], structural equation model analysis was performed to verify the research model. Moreover, the Phantom Model Approach was used to test the indirect effect of each mediating variable. This approach was proposed by Macho and Ledermann [41] as a bootstrapping method by adding phantom variables into the model to each mediating effect in a multiple mediation model.

## 3. Results

### 3.1. Descriptive Statistics

Table 3 shows the results of the normality of the variables measured in this study. As a result of the analysis, the normality was confirmed with the skewness (−0.267~0.097) and kurtosis (−0.873~0.890).

### 3.2. Correlations between the Measured Variables

The correlation analysis was performed to verify the relationship between major variables. As a result, as shown in Table 4, there were partial correlations between the sub-factors of the main variables. In addition, all coefficients were below 0.80, the standard for multicollinearity, indicating that there is no multicollinearity problem [42].

### 3.3. Testing the Measurement Model

**Figure 1:**
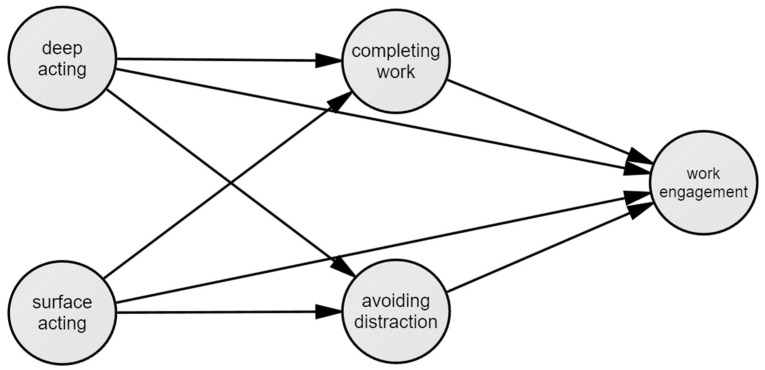
Measurement model.

According to the recommendation of Anderson and Gerbing [40], the measurement model was verified before the main analysis. As a result, the model fit was acceptable (*χ*^2^ = 188.771, df = 125, TLI = 0.967, CFI = 0.973, RMSEA = 0.051, SRMR = 0.0493), and all the standardized coefficients (*β*) were higher than 0.657, indicating that the fitness criteria suggested by Kline [42] were met. After establishing the statistical model as shown in Figure 1, structural equation model analysis was performed.

### 3.4. Structural Equation Model Analysis

Deep acting and surface acting, which are sub-factors of emotional labor, were set as exogenous variables (independent variables), and completing work and avoiding distraction, which is sub-factors presenteeism, were set as endogenous variables (mediators), and work engagement was set as the dependent variable after defining as a single factor through item parceling.

The detailed results of each path are shown in Table 5. As a result, the deep acting of disability sports coaches negatively predicted completing work (*β* = −0.409, *t* = −5.655 ***) and avoiding distraction (*β =* −0.311, *t* = −3.917 ***), while it positively predicted work engagement (*β* = 0.217, *t* = 3.307 ***), and all the paths were statistically significant. The path between coaches’ surface acting and completing work was found to be statistically significant (*β* = −0.26, *t* = −3.67 ***), and surface acting positively predicted work engagement (*β* = 0.168, *t* = 2.707 **), but the path between surface acting and avoiding distraction was not statistically significant. According to the result, it was found that completing work had a negative effect on work engagement, which was statistically significant (*β* = −0.549, *t* = −6.066 ***) but avoiding distraction did not have a statistically significant effect on coaches’ work engagement.

### 3.5. Mediating Effect of Presenteeism

The bootstrapping method was used to verify the indirect effect of the path from emotional labor (deep acting, surface acting) to work engagement via completing work in presenteeism. Repetition was performed 2000 times, and statistical significance was verified at the 95% confidence interval.

**Figure 2:**
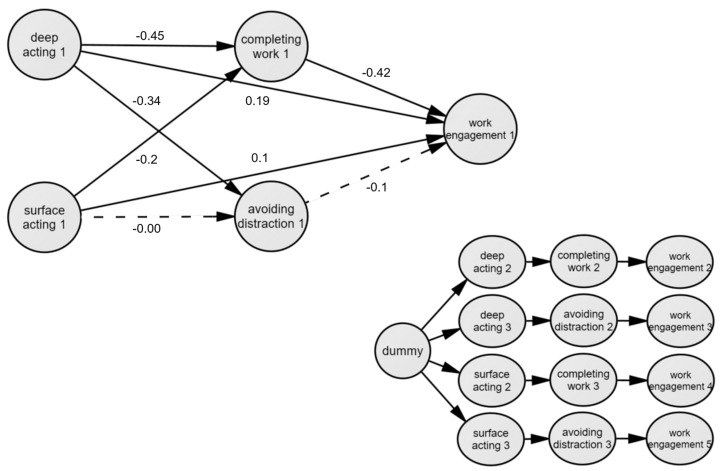
Mediation model using Phantom Model Approach.

As a result of analyzing the indirect effects of two mediators by applying the phantom model approach (Figure 2), the mediating effects of completing work in the relationship between deep acting and work engagement and between surface acting and work engagement were both statistically significant (Table 6).

## 4. Discussion

This study aims to investigate the relationship between emotional labor, presenteeism, and work engagement of coaches for disabled sports. To this end, deep acting and surface acting, which are sub-factors of emotional labor, were set as independent variables; completing work and avoiding distraction, which are sub-factors of presenteeism, were set as mediators; and work engagement was set as the dependent variable. A discussion of the main results follows.

First, deep acting of the coaches positively predicted their work engagement and this is in line with previous studies [7,14,15,16,17]. This means that the more coaches try to align their real emotions with the emotions demanded by the workplace, the greater their vigor, dedication, and absorption for their work with the athletes with disability. On the other hand, unlike the results of several previous studies, it was found that coaches’ surface acting also positively explained work engagement. This indicates that hiding their real emotions and expressing emotions required in the workplace can also positively related with work engagement. It could be interpreted as coaches feeling more engaged in their work, considering the effort to fabricate their emotions as a kind of work effort [20].

Second, the emotional labor of coaches for disability sports was found to influence presenteeism. Specifically, deep acting of emotional labor was found to reduce completing work and avoiding distraction of presenteeism. And the emotional labor was the result of presenteeism. The surface acting of emotional labor was shown to reduce completing work of presentism, but the correlation with avoiding distraction was not significant. For sports coaches, deep acting means controlling their emotions for their athletes [3,4,5]. Although this requires more psychological effort, it is reported that there is a positive correlation with job-related factors [5]. On the other hand, it is suggested that surface acting shows a positive relationship with negative factors such as burnout, which is a method in which the coach fabricates his/her emotions with false laughter or expressions for effective coaching [3,4,5]. Since this is an act that induces emotional exhaustion, it was expected to cause presenteeism, but in this study, it was found that surface acting can reduce completing work in emotional labor, and this result can be said to be contrary to previous studies [43]. However, according to another study, when the satisfaction of the customers increases due to the surface acting, the job satisfaction of the employee who provides the service may increase [44]. In other words, if the coaches participated in this study experienced an increase in satisfaction of the athletes with disability due to their surface acting, it can be thought that the completing work was rather reduced.

Third, completing work in presenteeism had a negative effect on coaches’ work engagement, but avoiding distraction did not have a statistically significant effect on work engagement. These results were found to support the studies that presenteeism is highly correlated with job-related variables [45,46]. What is noteworthy in this study is that completing work in presenteeism was found to decrease coaches’ work engagement, but avoiding distraction was not related to the engagement. In other words, the difficulty of performing a job even if a condition problem occurs reduces work engagement, but the attention problem itself caused by the condition problem does not affect engagement [46]. This is considered to have an adverse effect on both the disabled sports coaches and the athletes, as performing the job as a coach despite having health-related problems to the extent that it interferes with work negatively affects their vigor, dedication, and absorption related to coaching.

Finally, the result of mediating effect analysis, the indirect effects of completing work in presenteeism were both statistically significant in the relationship between deep acting and work engagement and between surface acting and work engagement. These results imply that deep acting and surface acting that constitute emotional labor ultimately function as variables that increase the work engagement of disability sports coaches. In other words, the emotional labor of coaches can increase job engagement by diminishing presenteeism. This result supports the existing research that deep acting increases positive job-related variables [5], and the possibility that surface acting can also be positively linked with job-related factors which are contrary to previous studies [43,47]. At the same time, this result emphasizes the role of completing work in presenteeism. In other words, it is interpreted that sports coaches both modulating or fabricating their emotions for the athlete can increase their vigor, dedication, and absorption to their work by raising the awareness that they can perform their tasks properly even in negative conditions. Contrary to the existing perception, the result of surface acting also lowering presenteeism and increasing work engagement suggests the possibility of another mediating variable such as emotional intelligence.

## 5. Conclusions

In the present study, it was found that the emotional labor of coaches for disability sports has a close correlation with the presenteeism and work engagement of coaches. In addition, there was a mediating effect of completing work in presenteeism between their emotional labor and work engagement. Thus, it is the most important implication that, emotional labor of coaches with disability in job performance, that is, efforts to align emotional demands with their own emotions or to just express the desired emotions, not only has a direct positive effect on their work engagement, but also has an indirect effect via diminishing presenteeism. In other words, since coaching disabled athletes is a highly emotionally demanding job, coaches need to control their emotions to become more engaged with their work, especially to put emotional efforts to reduce work losses due to presenteeism. This can be used as a practical implication for coaches with disability athletes to control their emotions for effective coaching such as improving performance and conditioning of athletes.

The limitations of the present study and suggestions for the following studies are as follows. First, most of the participants in this study were coaches with sufficient coaching experience for disabled athletes. This means that they may already have a high level of ability for emotional regulation, and it may indicate that emotional labor has only positive aspects in this study. Therefore, it can be said that it is meaningful to consider antecedent variables that can affect emotional labor, such as emotional intelligence, in future research.

In addition, this study was designed under the assumption that disability sports coaches had a higher emotional labor level than general sports coaches. However, since sufficient research results on this assumption have not been accumulated, it will be difficult for the implications of this study to be widely used. Therefore, it will be necessary to further expand the implications of this study by conducting follow-up studies with general sports coaches.

## Figures and Tables

**Table 1:** Characteristics of the participants.

Characteristics	Category	N	%
Gender	Men	137	69.2
	Women	61	30.8
Age	less than 30	30	15.2
	31~40 or less	77	38.9
	41~50 or less	66	33.3
	50 or more	26	12.6
Years of coaching	less than 3	45	22.7
	3~less than 5	84	42.4
	5~less than 7	35	17.6
	7 or more	34	17.2

N = the number of participants.

**Table 2:** Reliability and Validity of Constructs.

Latent Variables			Observed Variables	B	*β*	SE	*t*	AVE	C.R	*α*
Emotional Labor	Deep Acting	→	a1	1.000	0.898			0.937	0.984	0.905
		→	a2	1.067	0.893	0.058	18.2 ***			
		→	a3	0.913	0.807	0.063	14.5 ***			
		→	a4	0.955	0.777	0.070	13.6 ***			
	Surface Acting	→	a5	1.000	0.820			0.928	0.981	0.912
		→	a6	0.996	0.842	0.071	13.9 ***			
		→	a7	1.120	0.901	0.075	14.9 ***			
		→	a8	1.022	0.824	0.076	13.4 ***			
Presenteeism	Completing Work	→	b1	1.000	0.818			0.932	0.982	0.907
		→	b3	1.003	0.814	0.074	13.4 ***			
		→	b4	1.021	0.916	0.065	15.5 ***			
		→	b5	0.954	0.827	0.070	13.5 ***			
	Avoiding Distraction	→	b6	1.000	0.813			0.910	0.968	0.834
		→	b7	1.136	0.909	0.092	12.2 ***			
		→	b8	1.035	0.687	0.102	10.1 ***			
Work Engagement	Vigor	→	c1	1.000	0.729			0.895	0.971	0.829
		→	c2	1.162	0.891	0.095	12.31 ***			
		→	c3	0.993	0.825	0.091	10.82 ***			
		→	c4	0.640	0.559	0.085	7.45 ***			
	Dedication	→	c7	1.000	0.692			0.892	0.970	0.880
		→	c8	1.189	0.905	0.103	11.55 ***			
		→	c9	1.238	0.888	0.109	11.30 ***			
		→	c10	1.057	0.747	0.108	9.79 ***			
	Absorption	→	c12	1.000	0.653			0.835	0.953	0.838
		→	c14	1.337	0.656	0.140	5.56 ***			
		→	c16	1.737	0.783	0.183	6.13 ***			
		→	c17	1.828	0.839	0.105	5.98 ***			

*** *p* < 0.001.; B = estimates; *β* = standardized estimates; SE = standard error; *t* = t value; AVE = average variance extracted; C.R = composite reliability; α = Cronbach’s alpha.

**Table 3:** Test of normality.

Variables	M	SD	Skewness	SE	Kurtosis	SE
Deep Acting	3.71	0.677	−0.09	0.173	0.126	0.344
Surface Acting	2.87	1.041	0.013		−0.872	
Completing Work	2.44	0.806	0.093		−0.301	
Avoiding Distraction	2.69	0.843	−0.274		−0.574	
Vigor	3.18	0.761	−0.198		0.135	
Dedication	3.64	0.698	−0.261		0.895	
Absorption	3.39	0.700	0.115		0.541	

M = mean; SD = standard deviation; SE = standard error.

**Table 4:** Correlation Matrix.

	1	2	3	4	5	6	7
1. Deep Acting	1						
2. Surface Acting	0.177 *	1					
3. Completing Work	−0.400 **	−0.300 **	1				
4. Avoiding Distraction	−0.295 **	−0.010	0.459 **	1			
5. Vigor	0.437 **	0.258 **	−0.504 **	−0.379 **	1		
6. Dedication	0.414 **	0.383 **	−0.639 **	−0.376 **	0.629 **	1	
7. Absorption	0.345 **	0.116	−0.476 **	−0.222 **	0.530 **	0.577 **	1

* *p* < 0.05, ** *p* < 0.01.

**Table 5:** Structural equation model analysis.

Latent Variables			B	*β*	SE	*t*
Deep Acting	→	Completing Work	−0.453	−0.409	0.080	−5.655 ***
	→	Avoiding Distraction	−0.343	−0.311	0.088	−3.917 ***
	→	Work Engagement	0.185	0.217	0.056	3.307 ***
Surface Acting	→	Completing work	−0.198	−0.26	0.054	−3.67 ***
	→	Avoiding Distraction	−0.002	−0.03	0.059	−0.034
	→	Work Engagement	0.099	0.168	0.036	2.707 **
Completing Work	→	Work Engagement	−0.422	−0.549	0.07	−6.066 ***
Avoiding Distraction	→		−0.093	−0.12	0.055	−1.674

** *p* < 0.01, *** *p* < 0.001.; B = estimates; *β* = standardized estimates; SE = standard error; *t* = t value.

**Table 6:** Test of mediating effects.

Path of Mediating Effect					Indirect Effect	*p*
Deep Acting	→	Completing Work	** → **	Work Engagement	0.191	0.001
Surface Acting	→	Completing Work	→	Work Engagement	0.084	0.002
*χ*^2^ = 188.771, df = 125, TLI = 0.967, CFI = 0.973, RMSEA = 0.051, SRMR = 0.0493						

## Data Availability

The authors declare that all data and materials are available to be shared on a formal request.
